# Integrative Analysis of Flavoromics, Lipidomics, and Transcriptomics Reveals the Potential Mechanisms Underlying the Unique Meat Flavor of Jianli Pigs

**DOI:** 10.3390/foods14223838

**Published:** 2025-11-10

**Authors:** Tong Chen, Sujian Lu, Jiawei Zhou, Zhong Xu, Yue Feng, Mu Qiao, Dake Chen, Zipeng Li, Hua Sun, Xianwen Peng, Shuqi Mei, Junjing Wu

**Affiliations:** 1 Hubei Key Laboratory of Animal Embryo and Molecular Breeding, Institute of Animal Husbandry and Veterinary, Hubei Provincial Academy of Agricultural Sciences, Wuhan 430064, China; ctokay@163.com (T.C.); mqbetter@163.com (M.Q.);; 2 Hubei Hongshan Laboratory, Wuhan 430064, China

**Keywords:** JL pigs, flavor compounds, flavor precursors, lipid composition, transcriptome

## Abstract

Jianli pig (JL) is a representative Chinese local pig breed with a unique fruity flavor and excellent meat quality. However, the reasons for the unique fruity flavor of Jianli pork are still unclear. This study explored the potential genetic mechanisms by performing an integrative analysis of the flavoromics, lipidomics, and transcriptomics of the longissimus thoracis (LT) from JL pigs and Duroc × Landrace × Yorkshire pigs (DLY). The results indicated that the relative abundance of Acetic acid butyl ester and 3-Carene flavor compounds characterized by sweet and fruity aroma in JL pork was higher compared with DLY pigs. Lipidomics results showed that 16-carbon and 18-carbon fatty acids are important lipid precursors for the flavor of JL pork. Moreover, two clusters of functional genes correlated with 3-Carene (*DCHS2*, *NRXN1*, *JAKMIP3*, and *TRO*) and Acetic acid butyl ester (*WFIKKN2*, *CES3*, and *IYD*) were identified. This study enriched the limited understanding of the unique fruity flavor formation in JL pigs, provided a theoretical basis for the breeding of high-quality pig breeds, and the processing of flavorful meat products.

## 1. Introduction

Pork is an important component of meat consumption, and meat quality and flavor are important criteria for consumers to evaluate and choose meat. Previous studies have shown that Chinese local pig breeds such as Chinese black pig [1], Guangdong small-ear spotted pigs [2], Ningxiang pigs [3], Chalu black pigs [4], Jiaxing black pigs [5], Beijing black pig, and Laiwu black pigs [6] exhibit unique flavor characteristics to varying degrees compared with commercial pork. JL pigs are one of the famous local breeds in China, originating from Jianli County, Hubei Province, China. Due to its excellent meat quality, like tender texture and fruity aroma, it was presented as tribute pork to the capital of the Qing Dynasty in the sixteenth year of the Qianlong reign (1752) [7]. However, the molecular mechanisms and genetic basis underlying the fruity flavor formation of JL pigs remain unclear.

Flavor is a complex sensory impression formed by the combination of meat aroma and taste [8]. During meat processing and storage, volatile compounds are the main contributors to aroma, comprising low-molecular-weight chemicals in nature, such as pyrazines, aldehydes, acids, ketones, hydrocarbons, esters, alcohols, and nitrogen-containing and sulfur-containing compounds [1,9,10]. Non-volatile components are important contributors to flavor precursors (such as sugars, peptides, amino acids, inorganic salts, and organic acids) and mouthfeel (such as sweet, saltiness, bitterness, and sourness). Flavor precursors are the material basis for the formation of meat flavor quality. The degradation of flavor precursors (glycolysis, protein hydrolysis, lipid degradation, and thiamine degradation), lipid oxidation, Maillard reaction, and the interactions among the products of these processes ultimately form the unique flavor of meat products [11,12]. However, the molecular characteristics of flavor compounds in Jianli pigs remain to be explored.

Meat flavor quality is primarily affected by factors such as animal breed, gender, nutrition, storage and processing methods, and the composition of meat [13,14]. In fact, these factors ultimately influence meat flavor by altering the composition of meat. Flavor precursors are the material basis for the formation of meat flavor quality, including low-molecular-weight water-soluble compounds and lipids. Studies have shown that lipids are important precursors in the formation of flavor compounds, and differences in lipid content and composition significantly affect meat flavor [1,3,15,16]. On the one hand, during meat processing and storage, the oxidation of lipids and the Maillard reaction produce volatile flavor compounds such as aldehydes and ketones [17]. On the other hand, fatty acids generated from lipid hydrolysis produce esters with fruity and sweet flavors through esterification reactions, which are important flavor compounds in meat [1,15]. Research has demonstrated that intramuscular fat (IMF) content and lipid composition significantly influence meat flavor [16,18,19]. It is worth noting that a strong capacity for fat deposition is a prominent feature of JL pigs. However, whether the fruity flavor of JL pigs is related to their lipid composition remains unclear.

In this study, an integrative analysis of the flavoromics, lipidomics, and transcriptomics data of the LT tissues from JL and DLY pigs was performed to elucidate the unique fruity flavor compounds and lipid composition characteristics of JL pork. The study aimed to identify the key flavor compounds responsible for the distinctive flavor and the critical flavor precursors that influence pork flavor, decipher the molecular genetic mechanisms underlying the formation of flavor lipid precursors in JL pigs. This study will provide new insights into the mechanisms underlying the fruity flavor characteristics of pork from local pig breeds such as JL pigs, and offer a theoretical basis for the breeding of high-quality pig breeds and the processing of flavorful meat products.

## 2. Materials and Methods

### 2.1. Sample Information

Six JL and six DLY healthy castrated boars were acquired from Hubei Jinggong Breeding Co., Ltd. (Jianli, China). These pigs were managed under the same feeding program and conditions until 250 ± 5 days. After an overnight fast with unrestricted access to water, each pig was rendered unconscious through electrical stunning and subsequently exsanguinated. Approximately 20–30 g of longissimus thoracis (LT) muscle from the left side of each carcass was collected within 45 min post-slaughter, and flash-frozen in liquid nitrogen for omics analysis. All animal experiment was approved by the Animal Ethics and Welfare Committee of Hubei Academy of Agricultural Sciences (2024SC003).

### 2.2. Fat Deposition Performance Measurement

The average backfat thickness was measured at the first rib, last rib, and lumbar vertebra on the left side of the carcass based on the standards of the National Pork Producers Council (NPPC, 1991). The IMF content was measured via the Soxhlet petroleum ether extraction method as previously described [20].

### 2.3. Research Conditions for Flavoromics

Sample pretreatment and the flavoromics determination procedures were carried out according to previously described methods [21,22], with technical services provided by Suzhou BioNovoGene Biomedical Tech Co., Ltd., Suzhou, China. Raw data of flavor compounds were normalized using either total peak area or internal standard methods, and negative controls (quality control samples) were set during data filtering to exclude technical errors. Compound annotation was performed by retrieving the NIST 2020 database through Chroma TOF (v4.3x) software. Chemotaxonomic classification of identified compounds was conducted using the PubChem database (https://pubchem.ncbi.nlm.nih.gov/, accessed on 5 November 2025) and ClassyFire (v0.3.8) software, with the quantity and relative content of each category quantified. Sensory odor characteristics were analyzed and compared based on the FlavorDB database (https://cosylab.iiitd.edu.in/flavordb/, accessed on 5 November 2025). Multivariate statistical analysis employed Orthogonal Partial Least Squares Discriminant Analysis (OPLS-DA). Significance of inter-group differences was assessed using Student’s *t*-test or one-way ANOVA. Differential flavor compounds were screened according to the criteria: |log_2_FC| ≥ 1, *p* value < 0.05 and variable importance in projection VIP > 1.

### 2.4. Research Conditions for Lipidomics

Sample pretreatment and the lipidomics determination procedures were carried out according to previously described methods [2,3], with technical services provided by Beijing Biomarker Biotechnology Co., Ltd., Beijing, China. The raw data collected using MassLynx V4.2 is processed by Progenesis QI (v4.0) software for peak extraction, peak alignment and other data processing operations, based on the Progenesis QI software online METLIN database and Biomark’s self-built library for identification, negative controls (quality control samples) were set during data filtering to exclude technical errors, and at the same time, theoretical fragment identification and mass deviation All are within 100 ppm. Principal component analysis and Spearman correlation analysis were used to judge the repeatability of the samples within the group and the quality control samples. The identified compounds are searched for classification and pathway information in Lipidmaps databases. According to the grouping information, calculate and compare the difference multiples. *T* test was used to calculate the significance *p* value of each compound. The R language package (v1.6.2) ropls was used to perform OPLS-DA modeling, and 200 times permutation tests were performed to verify the reliability of the model. The VIP value of the model was calculated using multiple cross-validation. The method of combining the difference multiple. The screening criteria are *p* value < 0.05 and VIP > 1.

### 2.5. Research Conditions for Transcriptomics

Sample pretreatment and the transcriptomics determination procedures were performed according to previously described methods [2], with technical services provided by Wuhan Yingzi Gene Technology Co., Ltd., Wuhan, China. Raw sequencing data underwent quality control using Fastp (v0.23.2). Negative controls (quality control samples) were set during data filtering to exclude technical errors. Cleaned reads were aligned to the reference genome with HISAT2 (v2.2.1). Gene-level quantification across all samples was performed using featureCounts (v2.0.3). Differentially expressed genes (DEGs) were identified with either DESeq2 (v1.36.0) or edgeR (v3.38.4) R packages, applying thresholds of |log_2_FC| ≥ 1 and adjusted *p* < 0.05. Functional enrichment analysis of DEGs was conducted through KEGG and GO pathway analyses using the clusterProfiler R (v4.2.1) package.

### 2.6. Correlation Analysis

The correlation analysis was conducted in three main parts: (1) Correlation analysis between differentially abundant lipids and differentially abundant flavor compounds annotated as sweet and fruity. (2) Correlation analysis between the top 100 differentially expressed genes and differentially abundant lipids. (3) Correlation analysis between the top 100 differentially expressed genes and differentially abundant flavor compounds annotated as sweet and fruity. Signed correlation heatmaps with hierarchical clustering were generated using ggplot2 and pheatmap packages in R. Correlations were computed via Hmisc R (v5.1.2) packages with Pearson’s method, retaining pairs meeting: −0.80 ≥ R ≥ 0.80 and *p* < 0.05.

### 2.7. Statistical Analysis

The data were analyzed with Prism 8 software (GraphPad) and are presented as mean ± SDs. Two-tailed Student’s *t*-test was used to compare the differences between two groups, *p* < 0.05 was considered a significant difference (*), and *p* < 0.01 was classified as a highly significant difference (**).

## 3. Results

### 3.1. Comparative Analysis of Flavor Characteristics Between JL and DLY Pigs

**Figure 1:**
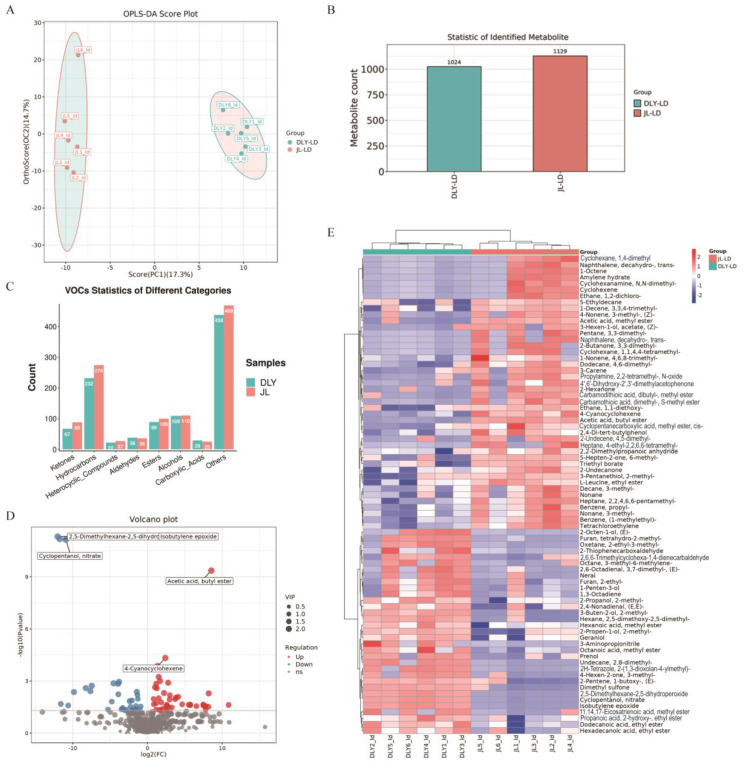
Comparison of flavor profiles in longissimus thoracis (LT) from Jianli (JL) and Duroc × Landrace × Yorkshire pigs (DLY). (**A**) Orthogonal Partial Least Squares–Discriminant Analysis (OPLS-DA) plot of flavor compounds in DLY and JL pigs. (**B**) Barplot of identified flavor compounds. (**C**) Barplot of classified and annotated flavor compounds. (**D**) Volcano plot of differences in flavor compounds between DLY and JL pigs. (**E**) Heatmap of differences in flavor compounds between DLY and JL pigs.

Flavoromics was performed to identify the differences in flavor compound composition in the LT muscle samples from JL and DLY pigs. The results showed significant differences in the composition of flavor compounds between JL and DLY pigs. A total of 1129 and 1024 flavor compounds were detected in JL and DLY pork, respectively, with JL pork showing a significant increase in the types of ketones, hydrocarbons, heterocyclic compounds, and esters compared with DLY pork (Figure 1A–C and Figure S1A,B). Compared with DLY pigs, 44 significantly upregulated and 33 significantly downregulated flavor compounds were identified in JL pork (Figure 1D,E).

**Figure 2:**
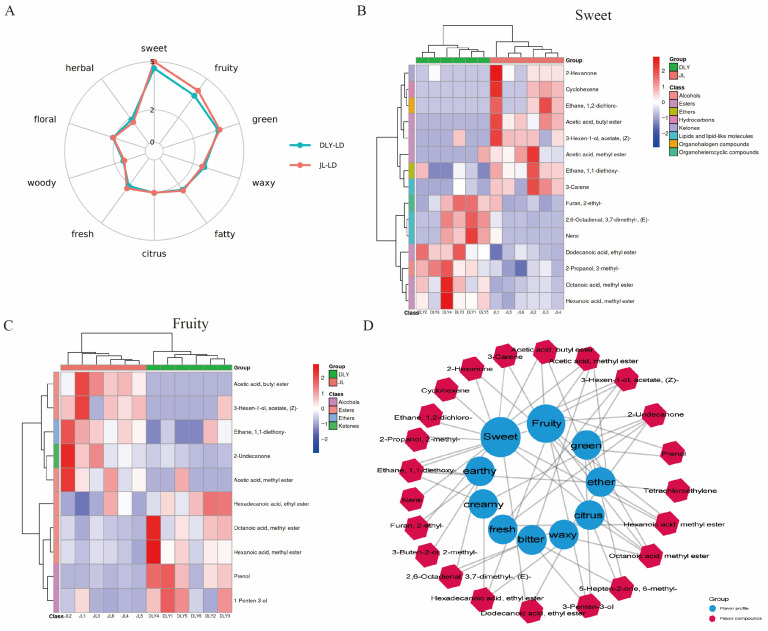
Functional annotation of differential flavor compounds in longissimus thoracis (LT) from Jianli (JL) and Duroc × Landrace × Yorkshire pigs (DLY). (**A**) Radar chart of sensory aroma characteristics of flavor compounds in DLY and JL pigs. (**B**,**C**) Heatmaps of relative abundance of sweet aroma (**B**) and fruity aroma (**C**) flavor compounds. (**D**) Correlation network between sensory aroma characteristics and flavor compounds.

Further analysis of the sensory flavor characteristics of flavor compounds revealed that JL pigs exhibited more pronounced sensory characteristics of sweet and fruity aroma compared with DLY pigs. The relative abundance analysis of flavor compounds revealed that the abundance of “Acetic acid butyl ester,” “3-Hexen-1-ol acetate,” “Acetic acid methyl ester,” “2-Hexanone,” and “3-Carene,” which were annotated as having sweet and fruity flavors, were significantly higher in JL pigs compared with DLY pigs (Figure 2A–C). However, the relative abundance of compounds with high relative flavor contribution (ROAV) values showed no significant differences between JL and DLY pigs (Figure S1C). The correlation network analysis between flavor compounds and sensory flavor characteristics further indicated that these compounds might be key components constituting the unique fruity flavor of JL pork (Figure 2D). The above results suggest that the different abundance in flavor compounds with low ROAV values are the main components mediating the flavor differences between JL and DLY pigs.

### 3.2. Comparative Analysis of Lipidomic Profiles Between JL and DLY Pigs

**Figure 3:**
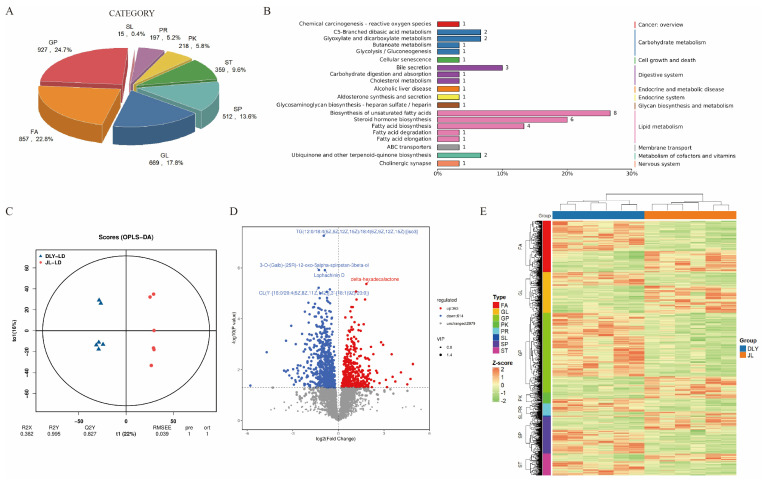
Comparison of lipidomic profiles in longissimus thoracis (LT)from Jianli (JL) and Duroc × Landrace × Yorkshire pigs (DLY). (**A**) Classification statistics of lipid composition. (**B**) Kyoto Encyclopedia of Genes and Genomes (KEGG) annotation results of identified lipids. (**C**) Principal Component Analysis (PCA) plot of lipid composition in DLY and JL pigs. (**D**) Volcano plot of differences in lipid composition between DLY and JL pigs. (**E**) Heatmap of clustered differences in lipid composition between DLY and JL pigs. Glycerophospholipids, GP; Fatty Acyls, FA; Glycerolipids, GL; Sphingolipids, SP; Sterol Lipids, ST; Polyketides, PK; Prenol Lipids, PR; Saccharolipids, SL.

The fat deposition-related carcass trait measurement results indicated that JL pigs exhibited a stronger lipid deposition capacity than DLY pigs, but there was no significant difference in IMF content between JL and DLY pigs (Table 1). Considering that lipids are important precursors of flavor compounds, we hypothesized that the differences in flavor compounds between JL and DLY pigs might be caused by differences in lipid composition. Lipidomics was performed to verify this hypothesis. Based on the functional definitions of lipids in the LIPID MAPS database, a total of 3754 lipid components were identified, and these lipid components were annotated into eight categories: Glycerophospholipids (GP), Fatty Acyls (FA), Glycerolipids (GL), Sphingolipids (SP), Sterol Lipids (ST), Polyketides (PK), Prenol Lipids (PR), and Saccharolipids (SL) (Figure 3A). These lipids were enriched in pathways related to fatty acid generation in the “Lipid metabolism” signaling pathway (Figure 3B). Consistent with the hypothesis, there were significant differences in the lipid composition profiles between JL and DLY pigs. 363 significantly upregulated and 614 significantly downregulated lipid components were identified in JL pork compared with DLY pigs (Figure 3C–E).

**Figure 4:**
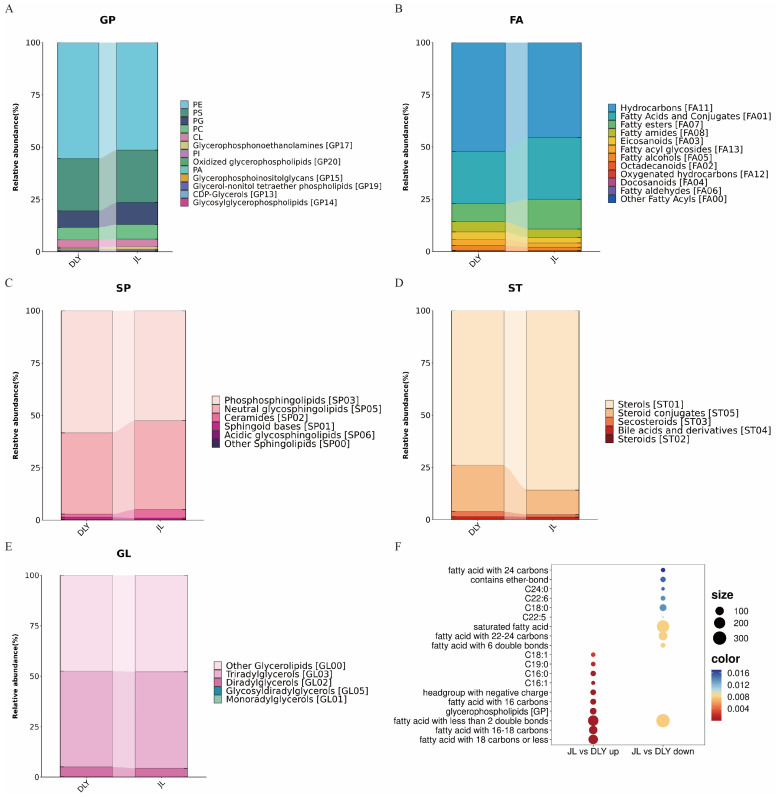
Annotation of differential lipids in longissimus thoracis (LT) from Jianli (JL) and Duroc × Landrace × Yorkshire pigs (DLY). (**A**–**E**) Stacked barplots of the composition of the top 5 differentially abundant lipid subclasses. (**F**) Dotplot of enriched lipid ontology terms for differentially abundant lipids. Glycerophospholipids, GP; Fatty Acyls, FA; Glycerolipids, GL; Sphingolipids, SP; Sterol Lipids, ST; Polyketides; Phosphatidylcholine, PC; Phosphatidylethanolamine, PE; Phosphatidylinositol, PI; Phosphatidylglycerol, PG; Phosphatidylcholine, PC; Cardiolipin, CL; Phosphatidic Acid, PA.

The relative abundance analysis of the top 5 differentially abundant lipid subclasses further clarified these detailed differences in lipid composition. The results showed that the relative abundance of phosphatidylethanol-amines (PE) decreased, while that of phosphatidylcholine (PC) and phosphatidylglycerol (PG) increased in the GP subclass of JL pigs. The relative abundance of Hydrocarbons decreased, while that of Fatty esters, Fatty acids and Conjugates increased in the FA subclass of JL pigs. The relative abundance of Steroid conjugates decreased, while that of Sterols increased in the ST subclass of JL pigs. The relative abundance of Phosphosphingolipids decreased, while that of Neutral glycosphingolipids and Ceramides increased in the SP subclass of JL pigs. There was no significant difference in the relative abundance of lipid components in the GL subclass between JL and DLY pigs (Figure 4A–E). Importantly, the lipid ontology enrichment analysis of differentially abundant lipids revealed distinct specificity in the high-abundance lipid categories between JL and DLY pigs. Specifically, the relative abundance of 16-carbon fatty acids and 18-carbon fatty acids significantly increased in JL pigs (Figure 4F). These results indicate significant differences in the fatty acid composition between JL and DLY pigs.

### 3.3. Correlation Analysis Identified the Precursor Lipids Associated with the Flavor of JL Pigs

**Figure 5:**
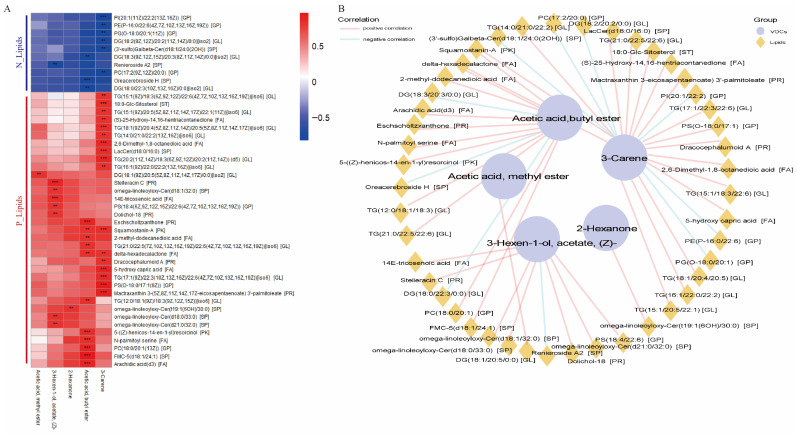
Correlation of differential lipids and flavor compounds between Jianli (JL) and Duroc × Landrace × Yorkshire pigs (DLY). (**A**) Correlation analysis of five key differentially abundant flavor compounds and differentially abundant lipids. (**B**) Multi-omics network of differentially abundant flavor compounds and lipids. ** *p* < 0.01; *** *p* < 0.001.

Based on the differences in the relative abundance of flavor compounds annotated as sweet and fruity in JL and DLY pigs, key flavor compounds such as “Acetic acid butyl ester,” “3-Hexen-1-ol acetate,” “Acetic acid methyl ester,” “2-Hexanone,” and “3-Carene” were identified. Correlation and association analyses between lipids and these flavor compounds were performed to further evaluate the contribution of lipids to flavor differences. The correlation analysis results showed that the lipid precursors significantly correlated with key flavor compounds were divided into two types: positively correlated lipids (P_Lipids) and negatively correlated lipids (N_Lipids). The lipids that were positively correlated with P_Lipids were mainly 16-carbon and 18-carbon fatty acids (Figure 5A,B). The results indicated that the contribution of different lipid precursors to flavor compounds was highly specific. Specifically, only DG(18:1(9Z)/20:5(5Z,8Z,11Z,14Z,17Z)) in the GL class of lipids was significantly positively correlated with “Acetic acid methyl ester”; only omega-linoleoyloxy-Cer(t19:1(6OH)/30:0) in the SP class of lipids was significantly positively correlated with “2-Hexanone”; “Acetic acid butyl ester” was significantly positively correlated with FA class lipids such as arachidic acid, N-palmitoyl serine, delta-hexadecalactone, and 2-methyl-dodecanedioic acid; “3-Carene” was correlated with eight major lipid components classes, including GP, FA, GL, SP, ST, PR, GP, and PK (Figure 5A,B). These results suggest that different types of lipids contribute differently to pork flavor compounds, and that 16-carbon fatty acids, 18-carbon fatty acids, and arachidonic acid may be important flavor precursors for the sweet and fruity aroma in JL pigs.

### 3.4. The Expression Patterns of Fatty Acid Metabolism-Related Genes Are Significantly Different Between JL and DLY Pigs

**Figure 6:**
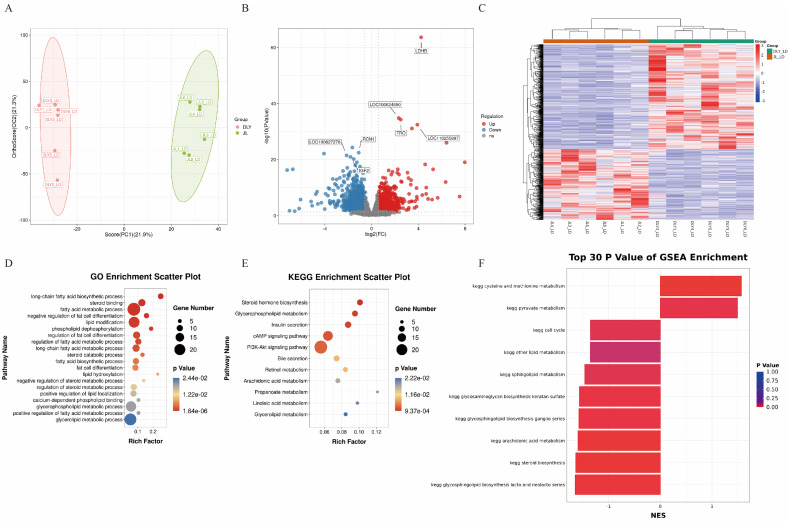
Comparison of transcriptomic profiles in longissimus thoracis (LT) from Jianli (JL) and Duroc × Landrace × Yorkshire pigs (DLY). (**A**) Principal Component Analysis (PCA) plot of gene expression in DLY and JL pigs. (**B**) Volcano plot of differentially expressed genes (DEGs) between DLY and JL pigs. (**C**) Heatmap of DEGs between DLY and JL pigs. (**D**) Dotplot of Gene Ontology (GO) functional enrichment analysis of DEGs between DLY and JL pigs. (**E**) Bubble chart of Kyoto Encyclopedia of Genes and Genomes (KEGG) enrichment analysis of DEGs between DLY and JL pigs. (**F**) Gene set enrichment analysis (GSEA) enrichment analysis of DEGs between DLY and JL pigs.

It is well known that gene regulation plays an important role in lipid metabolism. Therefore, transcriptomics was performed to elucidate the genetic regulatory mechanisms underlying the differences in lipidomic profiles between JL and DLY pigs. The transcriptomic results showed significant differences in gene expression profiles in the LT tissue of JL and DLY pigs. A total of 462 downregulated and 312 upregulated differentially expressed genes (DEGs) were identified in the LT of JL pigs (Figure 6A–C). Further functional enrichment analysis of DEGs showed that they were significantly enriched in fatty acid metabolism-related functional items such as “long-chain fatty acid biosynthetic process” and “fatty acid metabolic process” (Figure 6D–F and Figure S2A), which is consistent with the higher abundance of arachidonic acid in JL pigs. These results indicated significant differences in the expression patterns of fatty acid metabolism-related genes between JL and DLY pigs.

### 3.5. Integrative Analysis Revealed Key Regulatory Genes in the Flavor Formation of JL Pigs

**Figure 7:**
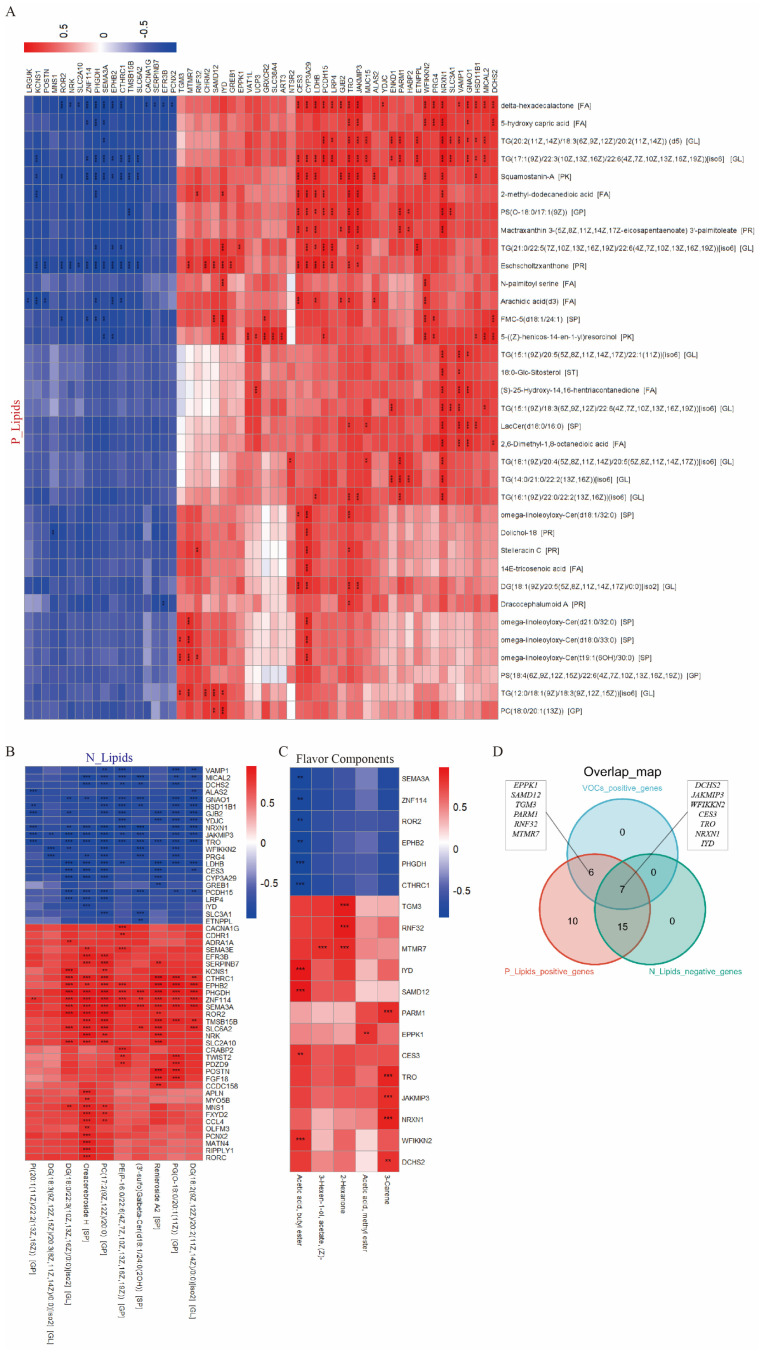
Integrative multi-omics analysis. (**A**) Correlation analysis of key positively correlated lipids (P_Lipids) and the top 100 DEGs. (**B**) Correlation analysis of key negatively correlated lipids (N_Lipids) and the top 100 DEGs. (**C**) Correlation analysis of the top 100 DEGs and key differentially abundant flavor compounds. (**D**) Overlap plot of key DEGs. ** *p* < 0.01; *** *p* < 0.001.

Integrative multi-omics correlation analysis was conducted to better understand the potential regulatory networks between genes, lipids, and flavor compounds. Firstly, the lipids contributing to the unique sweet and fruity aroma of JL pigs were divided into two categories: P_Lipids and N_Lipids (Figure 5A). Correlation analysis between these lipids and the top 100 differentially expressed genes identified 22 functional genes that may positively contribute to the key flavor precursor lipid components, which were significantly positively correlated with P_Lipids and significantly negatively correlated with N_Lipids (Figure 7A,B,D). Correlation analysis between the five key flavor compounds and the top 100 DEGs also identified 13 functional genes that may positively contribute to the flavor of JL pigs (Figure 7C,D). The overlap results of these functional genes showed that seven key functional genes positively contribute to both lipid flavor precursors and flavor compounds. These genes were divided into two clusters: those significantly correlated with 3-Carene (sweet aroma) (*DCHS2*, *NRXN1*, *JAKMIP3*, and *TRO*) and those significantly correlated with Acetic acid butyl ester (sweet, fruity aroma) (*WFIKKN2*, *CES3*, and *IYD*) (Figure 7C,D). These results suggest that genes such as *DCHS2*, *NRXN1*, and *JAKMIP3* may enhance the sweet aroma of pork by regulating the metabolism of 16-carbon and 18-carbon fatty acid flavor lipid precursors, while genes such as *WFIKKN2*, *CES3*, and *IYD* may enhance the sweet and fruity aroma of JL pork by regulating lipid metabolism.

## 4. Discussion

The flavor of meat is a combination of aroma mediated by volatile compounds and taste mediated by non-volatile compounds. Animal breed, gender, nutritional level, and meat processing methods are the main factors that influence meat flavor [8,13,14]. This study compared the differences in flavor compounds between JL and DLY pigs by integrating flavoromics, lipidomics, and transcriptomics. It was found that JL pigs showed a stronger sweet and fruity aroma compared with DLY pigs. Previous studies have shown that representative Chinese local pigs, such as Taoyuan black pig [20], Chalu black pig [21], Jiaxing black pig [5], Yunan black pig [1], Beijing black pig, Ningxiang black pig [3] and Wuzhishan pig [22], generally have better meat quality than lean-type pigs such as DLY. The contents of volatile flavor compounds with low thresholds, such as aldehydes, alcohols, ketones, and acids, are significantly higher in these local pigs compared with white lean-type pigs. Contrary to the conclusions of previous studies, this study found no significant differences in the volatile flavor compounds with high ROAV value (such as aldehydes) between JL and DLY pigs. However, significant differences were observed in the relative abundance of flavor compounds with low ROAV value. These results suggested that the unique flavor of JL pork is mainly influenced by the abundance of flavor compounds with low ROAV value, like esters. Importantly, the relative abundance of flavor compounds such as “Acetic acid butyl ester,” “3-Hexen-1-ol acetate,” “Acetic acid methyl ester,” “2-Hexanone,” and “3-Carene,” which showed sweet and fruity flavors, was significantly higher in JL pigs. These compounds have the potential to serve as signature flavor compounds for the unique flavor of JL pigs.

Lipids are the material basis for the formation of flavor quality. On the one hand, lipids are important precursors of meat flavor. On the other hand, lipid compounds reduce the volatility of most flavor compounds, thereby affecting the production of aromatic flavor compounds [8,23,24]. A flavor study indicated that the activity of fat-degrading enzymes increased after 7 days of beef processing, accompanied by a significant increase in the number of flavor compounds produced from fatty acid degradation [25]. Numerous studies have confirmed that the composition of fatty acids (such as PC and PE) and the degree of polyunsaturation significantly affect the abundance of flavor compounds produced after fatty acid oxidation [19,26,27]. Consistently, the carcass measurement results showed that JL pigs exhibited a stronger lipid deposition capacity than DLY pigs, although there was no significant difference in IMF content. Lipidomics results showed significant differences in lipid composition and fatty acid saturation between JL and DLY pigs. The relative abundance of fatty esters, PG, and PC was significantly higher in JL pigs than in DLY pigs. These relatively high-abundance lipids were mainly composed of saturated or monounsaturated 16-carbon and 18-carbon fatty acids (C16:1, C16:0, C18:1). Studies have shown that PC and PE play a crucial role in the formation of key aromatic compounds in meat products such as chicken and pork [19,26]. The low melting point of oleic acid (C18:1) can promote the rapid melting of fat in the mouth. As a key flavor precursor, its oxidation produces volatile compounds such as hexanal and octanal, which can significantly enhance the sweet, fruity, and fatty aroma of meat, enriching the flavor profile [28]. The downregulation of stearic acid (C18:0) effectively alleviates the hardening and greasiness caused by high melting point fatty acids [29]. These results indicate that the high abundance of 16-carbon and 18-carbon fatty acids (C16-C18) in JL pigs, which metabolize to produce flavor compounds, make significant contributions to the flavor characteristics of JL pigs.

The above results demonstrate that differences in lipid composition in JL pigs may be a key factor in forming its unique fruity flavor characteristics, but the molecular regulatory mechanisms underlying these differences in lipid composition remain unclear. Genes play an important regulatory role in lipid metabolism and composition. Previous studies have identified a series of key genes involved in the regulation of lipid composition, including *ACACA*, *FASN*, *FABP4*, *SCD1*, and *PPARγ*, through the integration of lipidomics and genome-wide association studies [30,31,32]. This study compared the gene expression profiles of JL and DLY pigs, clarified that genes related to fatty acid metabolism and associated signaling pathways are differentially expressed in JL pigs, and suggested that gene expression is involved in regulating the flavor compounds and lipid composition in JL pigs. Further integrative analysis of flavoromics, lipidomics, and transcriptomics identified two clusters of seven key functional genes significantly correlated with 3-Carene (sweet aroma) (*DCHS2*, *NRXN1*, *JAKMIP3*, and *TRO*) and Acetic acid butyl ester (sweet, fruity aroma) (*WFIKKN2*, *CES3*, and *IYD*). Based on the two clusters, the lipids significantly correlated with *TRO*, *JAKMIP3*, and *NRXN1,* mainly include TG (18:3) and PS (18:0); the lipids significantly correlated with *CES* and *WFIKKN2,* mainly include arachidonic acid, a known important flavor precursor. Studies have shown that TG (18:3) and PS (18:0) are important lipid precursors of key aromatic flavor compounds in fried chicken and pork [26,33]. *JAKMIP3* (also known as *NECC2*) is a gene highly expressed in secretory tissues such as adipose tissue. The protein it encodes has been shown to be located on the cell surface and is involved in regulating adipocyte differentiation and triglyceride hydrolysis metabolism through the insulin signaling pathway [34,35]. *WFIKKN2* is highly expressed in muscle tissue, positively correlated with body mass index [36] and involved in regulating adipocyte differentiation [37]. *CES3* encodes carboxylesterase, which is involved in the metabolism of fatty acyls and cholesterol esters. Numerous studies have shown that *CES3* is involved in regulating lipid droplet homeostasis [38,39,40,41,42]. In summary, this study indicated that genes such as *DCHS2*, *NRXN1*, *JAKMIP3, WFIKKN2*, *CES3*, and *IYD* may enhance the sweet and fruity aroma of pork by regulating lipid metabolism. However, the identification of these two clusters of genes is based on correlation predictions from flavoromics, lipidomics, and transcriptomics results. Validation through multi-breed data collection and gene function exploration at the cellular level in a testable model will be an important direction for future research.

Although this study has elucidated the potential molecular regulatory networks underlying the unique fruity flavor formation in JL pigs from the perspective of gene regulation of lipid composition and its impact on flavor compound formation by integrating flavoromics, lipidomics, and transcriptomics, providing new insights and a theoretical basis for the breeding of high-quality pig breeds and the processing of flavorful meat products, there are still some limitations. Current studies indicate that meat flavor precursors include low-molecular-weight water-soluble compounds (such as sugars, amino acids, and thiamine) and lipids. The Maillard reaction between reducing sugars and amino acids produces furans, thiophenes, and pyrazines, which are key flavor compounds in the formation of meat flavor [24,43]. Sulfur-containing amino acids, such as cysteine and methionine, can form volatile sulfur compounds with meaty aromas during the Maillard reaction [44]. Amino acids such as glutamate can enhance the umami taste of meat [45]. Glucose and xylose are also important components of the Maillard reaction. Studies have shown that high temperatures (above 100 °C) can significantly enhance the meaty aroma produced by the Maillard reaction between xylose and chicken peptides [45]. Pentoses and hexoses form different intermediates in the reaction, which in turn affect the final flavor [46]. This study has focused on the impact of lipid composition in JL pigs on flavor compound formation. Whether amino acids and sugars, which are part of the flavor precursors, are involved in regulating the unique fruity flavor formation in JL pigs remains to be further investigated. Moreover, the sample size (*n* = 6) involved in this study is somewhat insufficient. As previously mentioned, Animal breed, gender, nutritional level, and meat processing methods are the main factors that influence meat flavor [8,13,14]. Therefore, future studies need to incorporate more samples, breeds, and other publicly available omics data for comprehensive analysis, and ultimately validate the relevant results and conclusions through feasible models.

## 5. Conclusions

In summary, this study compared the differences in flavor compounds between JL and DLY pigs through flavoromics and identified components such as “Acetic acid butyl ester,” “3-Hexen-1-ol acetate,” “Acetic acid methyl ester,” “2-Hexanone,” and “3-Carene,” which have sweet and fruity flavors, as key flavor compounds in JL pigs. Lipidomics results showed that 16-carbon and 18-carbon fatty acids are important lipid precursors for the flavor of JL pork. Further integrated analysis constructed the following molecular regulatory networks: (A) genes such as *DCHS2*, *NRXN1*, and *JAKMIP3* enhance the sweet aroma of pork by regulating the metabolism of 16-carbon and 18-carbon fatty acid flavor lipid precursors; (B) genes such as *WFIKKN2*, *CES3*, and *IYD* enhance the sweet and fruity aroma of JL pork by regulating the metabolism of flavor lipid precursors. This study will provide new insights into the mechanisms underlying the unique flavor formation in local pigs, guiding new directions for high-quality pig breeding, pig husbandry management, and pork processing, and offering a theoretical basis for these areas.

## Figures and Tables

**Table 1:** Measurement results of fat deposition-related carcass traits in Jianli (JL) and Duroc × Landrace × Yorkshire pigs (DLY) (data are expressed as the mean ± SD, *n* = 6).

Items	DLY	JL	*p* Value
Carcass fat percentage (%)	19.33 ± 2.79	35.48 ± 3.60 **	0.00
Average backfat thickness (mm)	25.67 ± 6.11	34.86 ± 6.15 *	0.026
IMF content (%)	2.63 ± 0.61	3.15 ± 0.97	0.29

Notes: Intramuscular Fat Content, IMF; * *p* < 0.05; ** *p* < 0.01.

## Data Availability

The original contributions presented in the study are included in the article/Supplementary Material, further inquiries can be directed to the corresponding authors.

## Supplementary Materials

The following supporting information can be downloaded at: https://www.mdpi.com/article/10.3390/foods14223838/s1, Figure S1: Comparison of flavor profiles in LT between JL and DLY Pigs; Figure S2: Comparison of transcriptomic profiles in LT between JL and DLY Pigs.

## References

[1] Zhao J., Wang M., Xie J., Zhao M., Hou L., Liang J., Wang S., Cheng J. (2017). Volatile flavor constituents in the pork broth of black-pig. Food Chem..

[2] Wang X., Xu R., Tong X., Zeng J., Chen M., Lin Z., Cai S., Chen Y., Mo D. (2022). Characterization of different meat flavor compounds in Guangdong small-ear spotted and Yorkshire pork using two-dimensional gas chromatography–time-of-flight mass spectrometry and multi-omics. LWT.

[3] Wang Q., Gao H., Fu Y., Chen Y., Song G., Jin Z., Zhang Y., Yin J., Yin Y., Xu K. (2024). Comprehensive characterization of the differences in metabolites, lipids, and volatile flavor compounds between Ningxiang and Berkshire pigs using multi-omics techniques. Food Chem..

[4] Zhang Y., Zhang Y., Li H., Guo T., Jia J., Zhang P., Wang L., Xia N., Qian Q., Peng H. (2022). Comparison of Nutrition and Flavor Characteristics of Five Breeds of Pork in China. Foods.

[5] Chen Q., Zhang W., Xiao L., Sun Q., Wu F., Liu G., Wang Y., Pan Y., Wang Q., Zhang J. (2023). Multi-Omics Reveals the Effect of Crossbreeding on Some Precursors of Flavor and Nutritional Quality of Pork. Foods.

[6] Wu W., Zhan J., Tang X., Li T., Duan S. (2022). Characterization and identification of pork flavor compounds and their precursors in Chinese indigenous pig breeds by volatile profiling and multivariate analysis. Food Chem..

[7] Xu Z., Wu J., Zhang Y., Qiao M., Zhou J., Feng Y., Li Z., Sun H., Lin R., Song Z. (2024). Genome-wide detection of selection signatures in Jianli pigs reveals novel cis-regulatory haplotype in EDNRB associated with two-end black coat color. BMC Genom..

[8] Khan M.I., Jo C., Tariq M.R. (2015). Meat flavor precursors and factors influencing flavor precursors—A systematic review. Meat Sci..

[9] Chen G., Su Y., He L., Wu H., Shui S. (2019). Analysis of volatile compounds in pork from four different pig breeds using headspace solid-phase micro-extraction/gas chromatography-mass spectrometry. Food Sci. Nutr..

[10] Frank D., Appelqvist I., Piyasiri U., Delahunty C. (2012). In vitro measurement of volatile release in model lipid emulsions using proton transfer reaction mass spectrometry. J. Agric. Food Chem..

[11] Aaslyng M.D., Meinert L. (2017). Meat flavour in pork and beef—From animal to meal. Meat Sci..

[12] Sun A., Wu W., Soladoye O.P., Aluko R.E., Bak K.H., Fu Y., Zhang Y. (2022). Maillard reaction of food-derived peptides as a potential route to generate meat flavor compounds: A review. Food Res Int..

[13] Franco D., Crecente S., Vazquez J.A., Gómez M., Lorenzo J.M. (2013). Effect of cross breeding and amount of finishing diet on growth parameters, carcass and meat composition of foals slaughtered at 15 months of age. Meat Sci..

[14] Franco D., Rodriguez E., Purrinos L., Crecente S., Bermúdez R., Lorenzo J.M. (2011). Meat quality of “Galician Mountain” foals breed. Effect of sex, slaughter age and livestock production system. Meat Sci..

[15] Wang J., Yan Y., Peng X., Gao X., Luo Q., Luo Z., Wang K., Liu X. (2025). Linking lipidomics to meat quality: A review on texture and flavor in livestock and poultry. Food Chem..

[16] Huang Y., Zhou L., Zhang J., Liu X., Zhang Y., Cai L., Zhang W., Cui L., Yang J., Ji J. (2020). A large-scale comparison of meat quality and intramuscular fatty acid composition among three Chinese indigenous pig breeds. Meat Sci..

[17] Shahidi F., Hossain A. (2022). Role of Lipids in Food Flavor Generation. Molecules.

[18] Liu J., Huang F., Han D., Xu Y., Shen S., Luan Y., Yang P., Zhang C., Blecker C. (2025). Elucidation of potential lipid precursors and formation pathways for the warmed-over flavor (WOF) in precooked Chinese stewed beef through lipid oxidation mechanisms. Food Chem..

[19] Liu H., Ma Q., Xing J., Li P., Gao P., Hamid N., Wang Z., Wang P., Gong H. (2024). Exploring the formation and retention of aroma compounds in ready-to-eat roasted pork from four thermal methods: A lipidomics and heat transfer analysis. Food Chem..

[20] Shi H., Chen S., Zhou W., Xu J., Yang Z., Guo L., Li Q., Guo Q., Duan Y., Li J. (2025). A Comprehensive Characterization of the Differences in Meat Quality, Nonvolatile and Volatile Flavor Substances Between Taoyuan Black and Duroc Pigs. Foods.

[21] Zhang Y., Diao Y., Raza S.H.A., Huang J., Wang H., Tu W., Zhang J., Zhou J., Tan Y. (2025). Flavor characterization of pork cuts in Chalu black pigs using multi-omics analysis. Meat Sci..

[22] Tu T., Wu W., Tang X., Ge Q., Zhan J. (2021). Screening out important substances for distinguishing Chinese indigenous pork and hybrid pork and identifying different pork muscles by analyzing the fatty acid and nucleotide contents. Food Chem..

[23] Lee S., Jo K., Park M.K., Choi Y.-S., Jung S. (2025). Role of lipids in beef flavor development: A review of research from the past 20 years. Food Chem..

[24] Sohail A., Al-Dalali S., Wang J., Xie J., Shakoor A., Asimi S., Shah H., Patil P. (2022). Aroma compounds identified in cooked meat: A review. Food Res. Int..

[25] Gorraiz C., Beriain M.J., Chasco J., Insausti K. (2002). Effect of Aging Time on Volatile Compounds, Odor, and Flavor of Cooked Beef from Pirenaica and Friesian Bulls and Heifers. J. Food Sci..

[26] Liu H., Liu D., Suleman R., Gao P., Li P., Xing J., Ma Q., Hamid N., Wang P., Gong H. (2023). Understanding the role of lipids in aroma formation of circulating non-fried roasted chicken using UHPLC-HRMS-based lipidomics and heat transfer analysis. Food Res. Int..

[27] Elmore J.S., Mottram D.S., Enser M., Wood J.D. (1999). Effect of the polyunsaturated fatty acid composition of beef muscle on the profile of aroma volatiles. J. Agric. Food Chem..

[28] Navarro M., Dunshea F.R., Lisle A., Roura E. (2021). Feeding a high oleic acid (C18:1) diet improves pleasing flavor attributes in pork. Food Chem..

[29] Rodriguez E.E., Hamblen H., Leal-Gutierrez J.D., Carr C., Scheffler T., Scheffler J.M., Mateescu R.G. (2024). Exploring the impact of fatty acid composition on carcass and meat quality in Bos taurus indicus influenced cattle. J. Anim. Sci..

[30] Xiong L., Pei J., Wang X., Guo S., Guo X., Yan P. (2022). Effect of Lipids in Yak Muscle under Different Feeding Systems on Meat Quality Based on Untargeted Lipidomics. Animals.

[31] Li X., Wang Y., Guo J., Zhong T., Li L., Zhang H., Wang L. (2018). Identification and expression patterns of adipokine genes during adipocyte differentiation in the Tibetan goat (*Capra hircus*). Gene.

[32] Lin C., Dong Z., Song J., Wang S., Yang Y., Li H., Feng Z., Pei Y. (2023). Differences in histomorphology and expression of key lipid regulated genes of four adipose tissues from Tibetan pigs. PeerJ.

[33] Li J., Zhang Y., Zhang R., Yang R., Ma Q., Wang Z., Li P., Xing J., Gao P., Liu H. (2024). Unraveling the formation mechanism of aroma compounds in pork during air frying using UHPLC-HRMS and Orbitrap Exploris GC-MS. Food Res. Int..

[34] Zhu Y., Mao H., Peng G., Zeng Q., Wei Q., Ruan J., Huang J. (2021). Effect of JAK-STAT pathway in regulation of fatty liver hemorrhagic syndrome in chickens. Anim. Biosci..

[35] Travez A., Rabanal-Ruiz Y., Lopez-Alcala J., Molero-Murillo L., Díaz-Ruiz A., Guzmán-Ruiz R., Catalán V., Rodríguez A., Frühbeck G., Tinahones F.J. (2018). The caveolae-associated coiled-coil protein, NECC2, regulates insulin signalling in Adipocytes. J. Cell Mol. Med..

[36] Zaghlool S.B., Sharma S., Molnar M., Matías-García P.R., Elhadad M.A., Waldenberger M., Peters A., Rathmann W., Graumann J., Gieger C. (2021). Revealing the role of the human blood plasma proteome in obesity using genetic drivers. Nat. Commun..

[37] Zhang J.S., Xu H.Y., Fang J.C., Yin B.Z., Wang B.B., Pang Z., Xia G.J. (2021). Integrated microRNA-mRNA analysis reveals the roles of microRNAs in the muscle fat metabolism of Yanbian cattle. Anim. Genet..

[38] Lian J., Wei E., Wang S.P., Quiroga A.D., Li L., Di Pardo A., van der Veen J., Sipione S., Mitchell G.A., Lehner R. (2012). Liver specific inactivation of carboxylesterase 3/triacylglycerol hydrolase decreases blood lipids without causing severe steatosis in mice. Hepatology.

[39] Lian J., Quiroga A.D., Li L., Lehner R. (2012). Ces3/TGH deficiency improves dyslipidemia and reduces atherosclerosis in Ldlr^−/−^ mice. Circ. Res..

[40] Lagrutta L.C., Layerenza J.P., Bronsoms S., Trejo S.A., Ves-Losada A. (2021). Nuclear-lipid-droplet proteome: Carboxylesterase as a nuclear lipase involved in lipid-droplet homeostasis. Heliyon.

[41] Sakai K., Igarashi M., Yamamuro D., Ohshiro T., Nagashima S., Takahashi M., Enkhtuvshin B., Sekiya M., Okazaki H., Osuga J.-I. (2014). Critical role of neutral cholesteryl ester hydrolase 1 in cholesteryl ester hydrolysis in murine macrophages. J. Lipid Res..

[42] Yang L., Li X., Tang H., Gao Z., Zhang K., Sun K. (2019). A Unique Role of Carboxylesterase 3 (Ces3) in beta-Adrenergic Signaling-Stimulated Thermogenesis. Diabetes.

[43] Shi B., Guo X., Liu H., Jiang K., Liu L., Yan N., Farag M.A., Liu L. (2024). Dissecting Maillard reaction production in fried foods: Formation mechanisms, sensory characteristic attribution, control strategy, and gut homeostasis regulation. Food Chem..

[44] Zhao J., Wang T., Xie J., Xiao Q.F., Du W.B., Wang Y.X. (2019). Meat flavor generation from different composition patterns of initial Maillard stage intermediates formed in heated cysteine-xylose-glycine reaction systems. Food Chem..

[45] Ma X., Yu M., Liu Z., Deng D., Cui Y., Tian Z., Wang G. (2020). Effect of amino acids and their derivatives on meat quality of finishing pigs. J. Food Sci. Technol..

[46] Sun A., Chen L., Wu W., Soladoye O.P., Zhang Y., Fu Y. (2023). The potential meat flavoring generated from Maillard reaction products of wheat gluten protein hydrolysates-xylose: Impacts of different thermal treatment temperatures on flavor. Food Res. Int..

