# Supplementary material for: Integrative Analysis of Flavoromics, Lipidomics, and Transcriptomics Reveals the Potential Mechanisms Underlying the Unique Meat Flavor of Jianli Pigs
Source: Foods. 2025 Nov 10;14(22):3838. doi: 10.3390/foods14223838 (PMC12650885; doi:10.3390/foods14223838)
Supplement: Supplementary file 1 [file foods-14-03838-s001.zip › foods-3956425-supplementary.pdf]

Supplemental materials

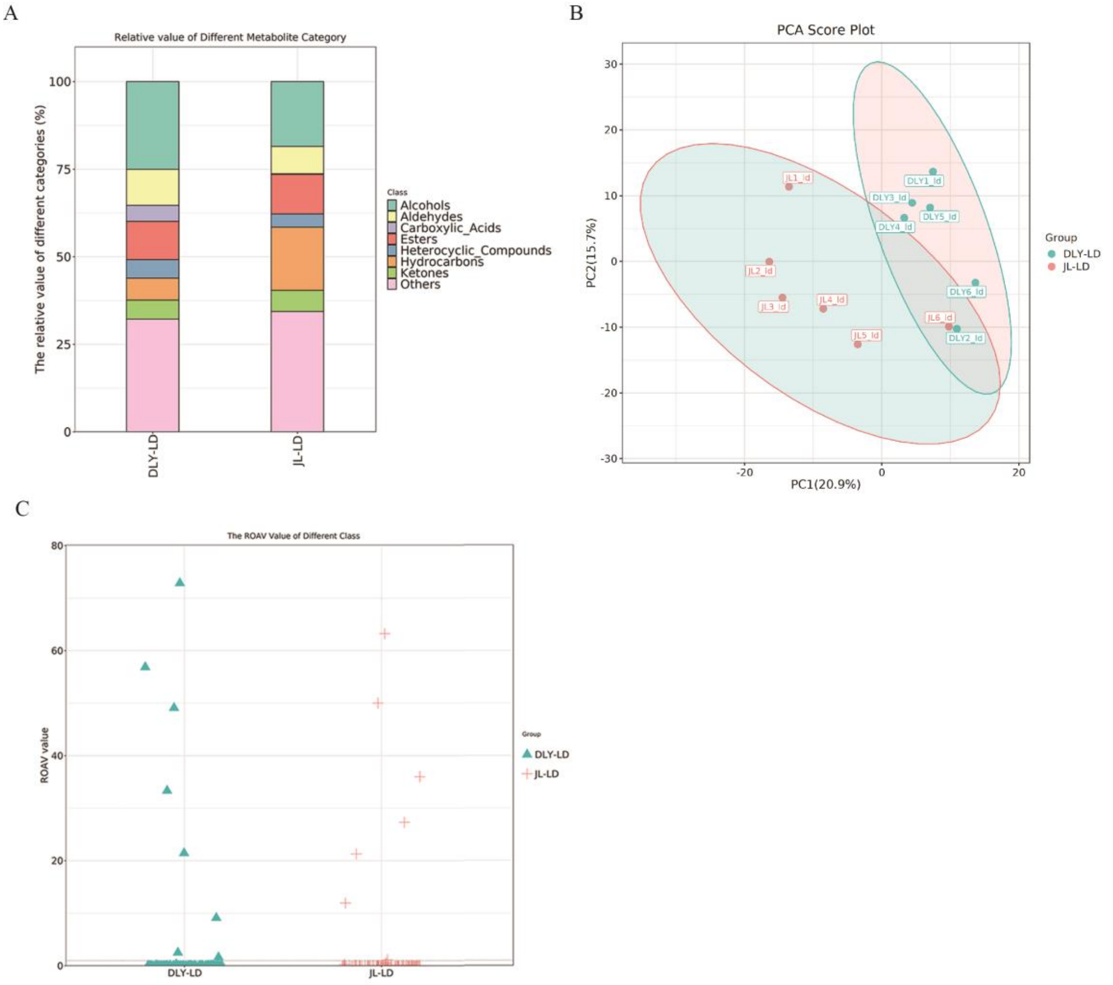

Supplemental Figure S1. Comparison of flavor profiles in LT between JL and DLY Pigs. (A) Stacked proportion chart of identified flavor compounds. (B) PCA plot of flavor compounds in JL and DLY pigs. (C) Volatility threshold chart of flavor compounds.

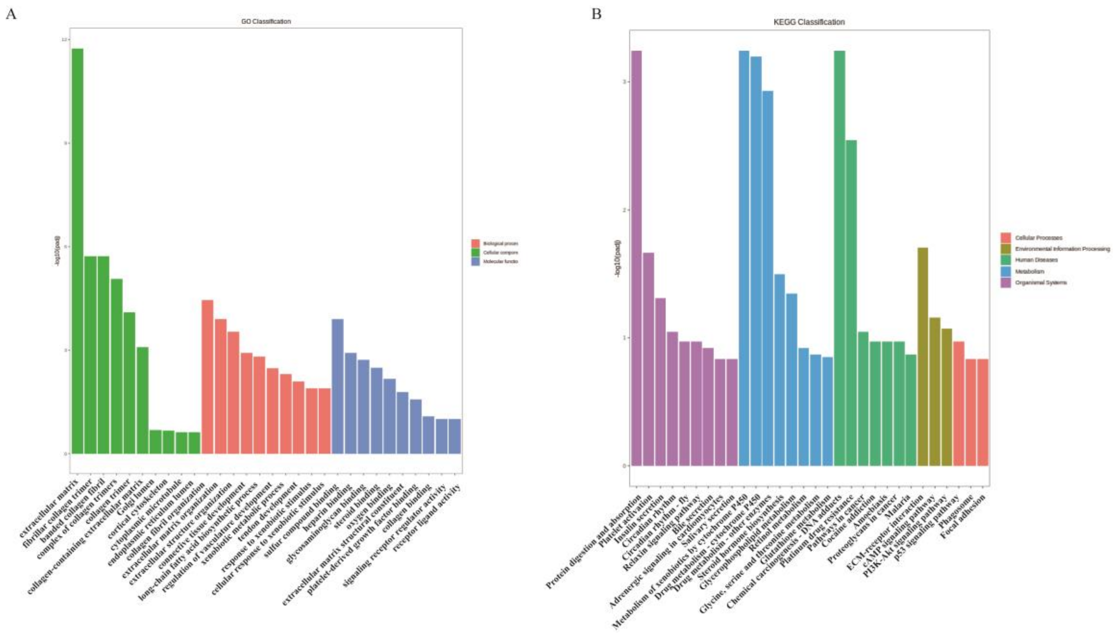

Supplemental Figure S2. Comparison of transcriptomic profiles in LT between JL and DLY Pigs. (A) Clustered bar plot of GO functional enrichment of differentially expressed genes. (B) Clustered bar plot of KEGG enrichment of differentially expressed genes.
